# Proteins Adsorbed during Intraoperative Hemoadsorption and Their In Vitro Effects on Endothelium

**DOI:** 10.3390/healthcare11030310

**Published:** 2023-01-19

**Authors:** Veronika Piskovatska, Alexander Navarrete Santos, Katrin Kalies, Edina Korca, Markus Stiller, Gábor Szabó, Andreas Simm, Kristin Wächter

**Affiliations:** 1 Department for Cardiac Surgery, University Hospital Halle (Saale), Martin Luther University Halle-Wittenberg, 06120 Halle (Saale), Germany; 2 Lipotype GmbH, 01307 Dresden, Germany; 3 Center for Medical Basic Research, Martin Luther University Halle-Wittenberg, 06120 Halle (Saale), Germany; 4 Clinic and Polyclinic of Internal Medicine III, University Hospital Halle (Saale), 06120 Halle (Saale), Germany

**Keywords:** hemoadsorption, blood purification, systemic inflammatory response, endothelium

## Abstract

(1) Background: Hemoadsorption is a method of blood purification with a wide spectrum of indications. Pre-emptive use of hemoadsorption in patients undergoing heart surgery with cardiopulmonary bypass is considered to reduce the risk of postoperative systemic inflammatory response syndrome. The current study aimed to identify the spectrum of blood proteins adsorbed on the polymer matrix of the CytoSorb hemoadsorption system and to investigate their influence on cultured endothelial cells in vitro. (2) Methods: Adsorbers used for intraoperative hemoadsorption were obtained from patients undergoing on-pump valve surgery in acute endocarditis. Proteins were extracted from the adsorbers, purified, identified with mass-spectrometry and applied to cultured human aortic endothelial cells. (3) Results: A broad range of blood proteins were identified in the material eluted from the CytoSorb adsorber. When added to cultured ECs, these protein extracts caused severe reduction in cell viability and migration. After 24 h exposure, transcriptional changes with up-regulation of multiple metabolic regulators were observed and verified on the protein level. Genes responsible for control of mitosis were significantly down-regulated. (4) Conclusions: In summary, our data reveal that intraoperative hemoadsorption allows broad spectrum removal of a wide range of molecules eliciting endothelial damage.

## 1. Introduction

About one-third of patients undergoing cardiac surgery with cardiopulmonary bypass (CPB) develop systemic inflammatory response syndrome (SIRS) [1]. CPB launches systemic inflammation through several mechanisms. Contact of blood with non-endothelial surfaces leads to cell and complement activation, and coagulation disturbances [2]. Additionally, damage to blood cells leads to the release of intracellular factors, instigating generalized inflammatory reaction and organ dysfunction [3]. Endothelium plays an important role in regulation of homeostasis and is a vulnerable target inevitably engaged into pathological processes initiated by CPB [4,5,6]. Endothelial cells produce a plethora of vasoactive substances, pro- and anti-coagulants, as well as inflammation mediators [7]. Intraoperative hemoadsorption has the potential to remove a plentitude of substances having unfavorable effects on endothelium, thus preventing endothelial damage and subsequent clinical outcomes of SIRS.

Current clinical evidence on the CytoSorb hemoadsorption device (CytoSorbents Corporation, Monmouth Junction, NJ, USA) demonstrates certain benefits of intraoperative hemoadsorption for patients undergoing valve surgery with CPB. Cases series analysis by Träger et al. suggests that the use of CytoSorb hemoadsorption reduces the need for vasopressors and reduces cytokine concentrations [8]. In a pilot-blinded randomized study comparing 19 patients assigned to hemoadsorption during elective CPB surgery versus 18 controls did not show any reduction in pro-inflammatory cytokine levels and no changes in their perioperative course [9]. A randomized controlled trial REFRESH I (REduction in FREe Hemoglobin) in patients undergoing cardiac surgery with prolonged cardiopulmonary bypass (CPB) demonstrated significant reduction in free hemoglobin and complement activation among patients allocated to hemoadsorption when compared to standard care [10]. A recent study, evaluating cytokine clearance in patients undergoing cardiac surgery, discovered no significant difference in concentrations of the main coagulation factors, except antithrombin and factor II, that were significantly decreased after the hemoadsorption [11]. Another study shows reduced incidence of post-operative sepsis and sepsis-related mortality in patients with native mitral valve endocarditis if they were allocated to hemoadsorption during valve surgery [12]. There is an ongoing discussion about whether CytoSorb blood purification improves the clinical outcomes [13,14]. In the recent REMOVE study, hemoadsorption was shown to reduce plasma cytokines during surgery in patients with infective endocarditis but did not lead to any reduction in postoperative organ dysfunction [15].

Evidence from in vitro and clinical studies confirms that the use of CytoSorb hemoadsorption significantly reduces concentrations of pro- and anti-inflammatory cytokines (IL-1β, IL-2, IL-6, IL-8, TNF-α) [16]. Clinical trials, case reports and case series describe the elimination of other substances and metabolites, like bilirubin [17,18,19], myoglobin [20], and free hemoglobin [10], cell-free DNA [21], and antithrombotic drugs [22]. The full spectrum of substances adsorbed from circulation, the extent of removal and potential clinical impact of depletion are yet to be elucidated.

## 2. Materials and Methods

**Patients and hemoadsorber acquisition.** Patients have been enrolled in 2018–2019 into a clinical study REMOVE [15] in the heart surgery department of the University Hospital Halle (ClinicalTrials.gov (accessed on 18 September 2022) Identifier: NCT03266302). Patients with infective endocarditis (according to Duke Criteria) undergoing valve surgery with CPB, aged ≥18 years, who signed informed consent were included into the study. Intraoperative HF was performed with CytoSorb 300 mL devices (CytoSorbents Corporation, Princeton, NJ, USA), combined with a roller-pump CBP machine HLM S5 (LivaNova, London, UK). Devices were rinsed with 0.9% saline to remove blood cells, debris and blood proteins not adsorbed on the beads. Afterwards, each device was disassembled, and the polymer beads were stored in 5.0 mL Eppendorf tubes at −20 °C until protein extraction.

**Isolation of proteins adsorbed on a polymer matrix.** CytoSorb beads were packed into 0.8 mL centrifuge columns (Thermo Fisher Scientific, Waltham, MA, USA), the material was rinsed twice with 1 × PBS (pH 7.4) to remove blood cells, cellular debris and proteins that could have remained unbound on the polymer. Proteins were eluted with 500 µL of 1 × RIPA-buffer (0.5 M Tris-HCl, pH 7.4, 1.5 M NaCl, 2.5% deoxycholic acid, 10% NP-40, 10 mM EDTA (Sigma Aldrich, St. Louis, MO, USA)) and centrifuged at 11,800× *g* for 1 min. at RT. A pool of four different elution samples, obtained from four adsorbers, was used to treat endothelial cells. 

**SDS-PAGE of isolated proteins.** Denatured protein samples were loaded into 12-well Criterion TGX pre-casted 4–20% gel (Bio-Rad Laboratories, Hercules, CA, USA) and run at 200 V. Afterwards, gel was rinsed for 15 min in deionized water and subsequently stained with Colloidal Coomassie Blue (CCB) (containing: 0.02% Colloidal Coomassie Blue G250 (Bio-Rad Laboratories, Hercules, CA, USA), 5% aluminum sulfate (Applichem, Darmstadt, Germany), 10% ethanol (Sigma Aldrich, St. Louis, MO, USA) and 2% ortophosphoric acid (Sigma Aldrich, St. Louis, MO, USA))for 3 h at RT with gentle shaking, destained overnight in water. Protein bands were visualized using 700 nm wavelength detection in an Odyssey Clx reader (LI-COR Biosciences, Lincoln, NE, USA).

**LC-MS/MS identification of isolated proteins.** Denatured protein samples (20 µg per lane) were separated by SDS-PAGE as described above (leaving each second pocket empty). Gel was stained with CCB and destained vide supra. Protein bands were excised, gel pieces were washed with 100 mM ammonium bicarbonate, pH = 7.8 (AppliChem, Darmstadt, Germany) and dehydrated with acetonitrile (Biosolve Chimie SARL, Dieuze, France) and dried in a Speed Vacuum Concentrator at RT for 5 min. Reduction was performed using 5 mM tris-(2-carboxyethyl)-phosphin (Thermo Fisher Scientific, Waltham, MA, USA) in 100 mM ammonium bicarbonate, pH = 7.8, for 15 min at 37 °C. Gel pieces were washed with acetonitrile twice and then dried for 10 min in a speed vacuum concentrator. Alkylation was performed with 55 mM iodoacetamide (Sigma Aldrich, St. Louis, MO, USA) in 100 mM ammonium bicarbonate, pH = 7.8, for 20 min in the dark. Gel pieces were then washed with ammonium bicarbonate twice and thereafter with a solution of 50 mM ammonium bicarbonate and 50% acetonitrile at 37 °C twice for 30 min. In the next step, the gel pieces were dehydrated with acetonitrile twice and dried in a speed vacuum concentrator at RT for 5 min. Gel pieces were rehydrated with a solution containing 260 ng trypsin (Promega, Madison, WI, USA) in 200 µL of 50 mM ammonium bicarbonate (pH 7.8) at 4 °C for 20 min. Digestion was performed overnight at 37 °C. The supernatant was taken off and peptides subsequently extracted with 50 mM ammonium bicarbonate (pH = 7.8), with 50% acetonitrile, 0.1% formic acid (Biosolve Chimie SARL, Dieuze, France) and 75% acetonitrile, 0.1% formic acid. The obtained peptide solutions were frozen at −80 °C and dried in a speed vacuum concentrator at RT overnight. Samples were analyzed by LC-MS/MS using the EASY-nLCTM 1000 system coupled to an Orbitrap Fusion™ Tribrid™ Mass Spectrometer (both Thermo Fisher Scientific, Waltham, MA, USA) by the company Proteome Sciences R&D GmbH & Co. KG (Frankfurt, Germany). Dried peptides were re-suspended in 2% ACN with 0.1% FA and a solid-phase purification step was performed using ZipTips (Merck Millipore, Burlington, MA, USA)) according to manufacturer instructions. After that, re-suspended peptides were loaded onto a nanoViper C18 Acclaim PepMap 100 pre-column (Thermo Fisher Scientific, Waltham, MA, USA) and resolved using an increasing gradient of ACN in 0.1% formic acid through an analytical column (Thermo Fisher Scientific, Waltham, MA, USA). Peptide mass spectra were acquired throughout the entire chromatographic run (60 min) using a top-speed collision-induced dissociation (CID) combined with the higher collision-induced dissociation (HCD) method. Raw mass spectrometry data files were submitted to Proteome Discoverer (PD) v2.1 (Thermo Fisher Scientific, Waltham, MA, USA) using the Spectrum Files node. The Precursor Ions Area Detector node was included in the workflow to enable extraction of MS1 peak area (intensity) values suitable for further label-free quantification.

**Cell culture.** Human aortic endothelial cells (HAECs) (Cell Applications Inc., San Diego, CA, USA) were used as a model to reveal effects of plasma proteins, isolated from the matrix of the adsorber. Cells were cultured with complete ECGM2 growth medium (PromoCell, Heidelberg, Germany) containing 10% fetal calf serum (FCS) (Zellbiologische Produkte GmbH, Lonsee, Germany) and 1% penicillin-streptomycin (Sigma Aldrich, St. Louis, MO, USA) in an incubator at 37 °C and 5% CO_2_. HAECs used for the experiments were between passages 6 and 10 to avoid growth and division retardation due to cellular senescence.

**Cell growth and viability assay.** HAECs were seeded on 96-well plate (black, flat transparent bottom, Eppendorf). To ensure that the cells are at the phase of exponential growth, a seeding density 1 × 10^4^ cells per well was used. The HAECs were cultivated in complete ECGM2 medium with 10% FCS overnight. The next day, cells were treated with proteins eluted from the hemoadsorber matrix (0.125 µg/mL; 0.25 µg/mL; 0.5 µg/mL) and corresponding concentrations of human serum (Sigma Aldrich, St. Louis, MO, USA). 2 mM hydrogen peroxide (University pharmacy Halle, Halle (Saale), Germany) was used as a reference cytotoxicant. All treatment and control agents were dissolved in ECGM2 without FCS. After 24 h stimulation, 20 µL of Cell Titer Blue (CTB, Promega, Madison, WI, USA) was added to each well. Cells were incubated with CTB for 4 h at 37 °C, and fluorescence measurements at Ex/Em = 560/590 nm were performed with microplate reader Tecan M1000 i-Control (Tecan Group, Männedorf, Switzerland).

**Wound-healing assay.** HAECs were seeded onto Nunclon 24-well plate (Thermo Fisher Scientific, Waltham, MA, USA) at seeding density 5 × 10^4^ cells per well. After overnight cultivation, cells were confluent and ready for migration assay. Confluent cell surface was scratched using sterile SPLScratcher (SPL Life Sciences, Naechon-myeon, Republic of Korea) with 24-well pattern. After this, cells were washed and plasma proteins from elution and human serum were added to each well in FCS-free ECGM2. Cell migration was visualized and recorded with Biotek Cytation (Agilent Technologies, Santa Clara, CA, USA) for 24 h with image recording every 30 min. Image series were evaluated with Biotek Gen5 software (Agilent Technologies, Santa Clara, CA, USA); area of the scratch, cell density and cell area were automatically calculated at each time point.

**Microarrays.** HAECs were cultivated on 10 cm cell culture dishes (TPP) until confluence, then cells underwent 24 h treatment with 0.125 µg/mL elution, 0.125 µg/mL serum or FCS-free ECGM2 as described above. Total RNA was isolated from HAECs by the TRIzol-method. For this purpose, cells were washed with 1 × PBS solution (Thermo Fisher Scientific, Waltham, MA, USA) and harvested with TRIzol Reagent (Thermo Fisher Scientific, Waltham, MA, USA, 1 mL per 6-well). After adding 200 µL of chloroform (Sigma Aldrich, St. Louis, MO, USA), mixing and centrifugation (2000× *g*, 5 min.) upper phase was gently agitated with isopropanol (Sigma Aldrich, St. Louis, MO, USA, 1:1 *v*/*v*) and incubated one hour at room temperature. RNA was pelletized by centrifugation (14.000× *g*, 10 min, 4 °C) and pellet was washed three times with 80% ethanol. After pellet drying nuclease free water was added and RNA was stored at −20 °C, an additional purification step was included by using RNeasy MinElute Cleanup Kit, Quiagen, Venlo, The Netherlands) according to manufacturer instructions. To ensure quality and integrity for subsequent array analyses, RNA was dissected by Bioanalyzer (Agilent Technologies, Santa Clara, CA, USA). Only RNA with RIN values ≥8 was used for further array analysis. Detection of RNA was performed by microarray (Clariom™ D Assay, Thermo Fisher Scientific, Waltham,, MA, USA) as previously described [23]. Data calculation was performed with the Robin 2.0 software [24].

**Western blot analysis**. HAECs were cultivated on 10 cm cell culture dishes (Sigma Aldrich, St. Louis, MO, USA) until confluency, then cells underwent 24 h treatment with 0.125 µg/mL elution, 0.125 µg/mL serum or FCS-free ECGM2 as described above. Afterwards, cells were washed with PBS and lysed with 100µL of 1 × RIPA buffer (Merck Millipore, Burlington, MA, USA). Lysates were denatured with Laemmli buffer at 95 °C for 5 min. Samples were separated by SDS-PAGE. Proteins were transferred onto 0.45 µm nitrocellulose membrane (Merck Millipore, Burlington, MA, USA) and blocked with 5% BSA (Applichem, Darmstadt, Germany) in TBS (Carl Roth, Karlsruhe, Germany) for 1 h at RT; afterwards a solution of primary antibody in 5% BSA-TBS-T (TBS with 0.1% Tween-20 (Applichem, Darmstadt, Germany)) was added to the membrane, and incubation lasted overnight at 4 °C on the rocking platform. After the incubation with primary antibody, the membrane was washed with TBS-T (TBS with 0.1% Tween-20 (Applichem, Darmstadt, Germany)) three times (each wash 5 min.) and incubated with secondary antibodies (Li-Cor Biosciences, Lincoln, NE, USA) for 1 h at RT on the rocking platform. Antibodies, dilutions and incubation times are described in Supplemental Table S1. Finally, the membrane was washed with TBS-T three times (each wash 5 min.) and visualized with Odyssey Clx scanner (LI-COR Biosciences, Lincoln, NE, USA). Images from the scanner were quantified with Odyssey Imaging system. Signal intensities from protein targets were normalized to signal intensities from β-actin. Normalized signal intensity from untreated control was taken for 100%; a 2-tailed, paired T-test was used to determine the level of significance for the difference between normalized signal intensities.

## 3. Results

### 3.1. Spectrum of Blood Proteins Adsorbed on the CytoSorb Polymer Matrix

Protein concentration in the eluted samples ranged between 1.78 and 6.52 µg/µL, on average 3.93 µg/µL. When characterized by SDS-PAGE, samples were rich in different proteins with a dominant band in the MW range of about 70 kDa. Each patient sample had an individual protein pattern with variable bands in the 25–35 kDa MW range and between 100 and 130 kDa (Supplemental Figure S1). The majority (83; 65.4%) of the isolated and by-mass spectrometry-identified proteins were, as expected, in the molecular weight range below 60 kDa. A total of 15 (12%) of 127 identified proteins had MW above 100 kDa. The majority of proteins belonged to classic residential plasma proteins, namely enzyme inhibitors, hydrolases and transport proteins. A full list of the 127 proteins, identified in all eluted samples can be found in Supplemental Table S2.

### 3.2. Alteration in Endothelial Cells Functionality after Treatment with Eluted Protein Mixture

#### 3.2.1. Cell Viability Assay

**Figure 1:**
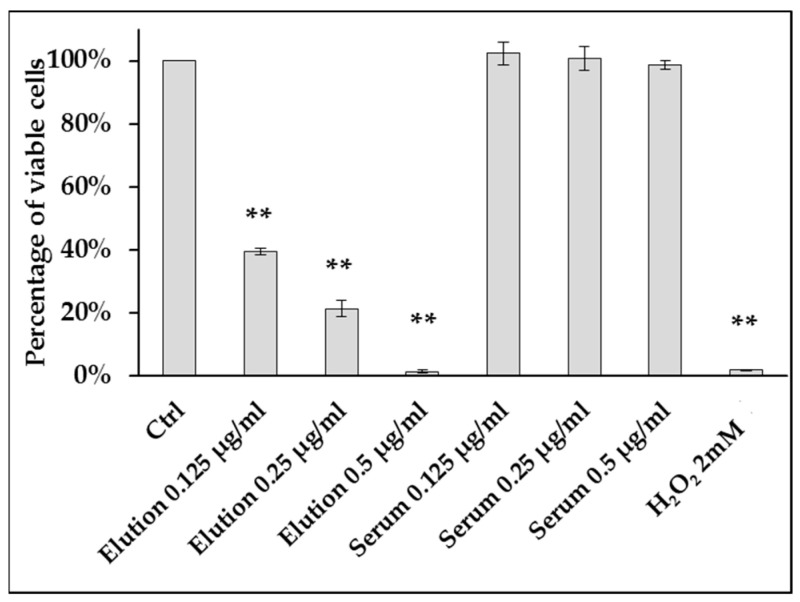
Changes in cell viability of HAECs treated with different concentrations of proteins isolated from polymer matrix (CTB-assay) (*n* = 3). Cells treated with increasing concentrations of eluted proteins (0.125 µg/mL, 0.25 µg/mL, 0.5 µg/mL) for 24 h, have demonstrated a dramatic drop in the rate of metabolism compared to untreated control (** *p* < 0.001, *t*-test).

Human Aortic Endothelial Cells (HAECs) cultured with protein mixture eluted from four adsorber devices demonstrated severe alterations in cell viability. Equivalent concentrations of serum did not produce significant changes in cell viability compared to untreated control. Cell viability was reduced in a concentration-dependent manner (by 60.45% and 78.51% in wells, treated with 0.125 µg/mL and 0.25 µg/mL of eluted proteins, correspondingly) after 24 h exposure. In wells exposed to 0.5 µg/mL of eluted proteins, reduction of cell metabolic activity was comparable to the addition of 2 mM hydrogen peroxide (Figure 1). No significant cell viability alterations in cells treated with corresponding concentrations of commercially available serum were observed.

#### 3.2.2. Wound-Healing Assay

**Figure 2:**
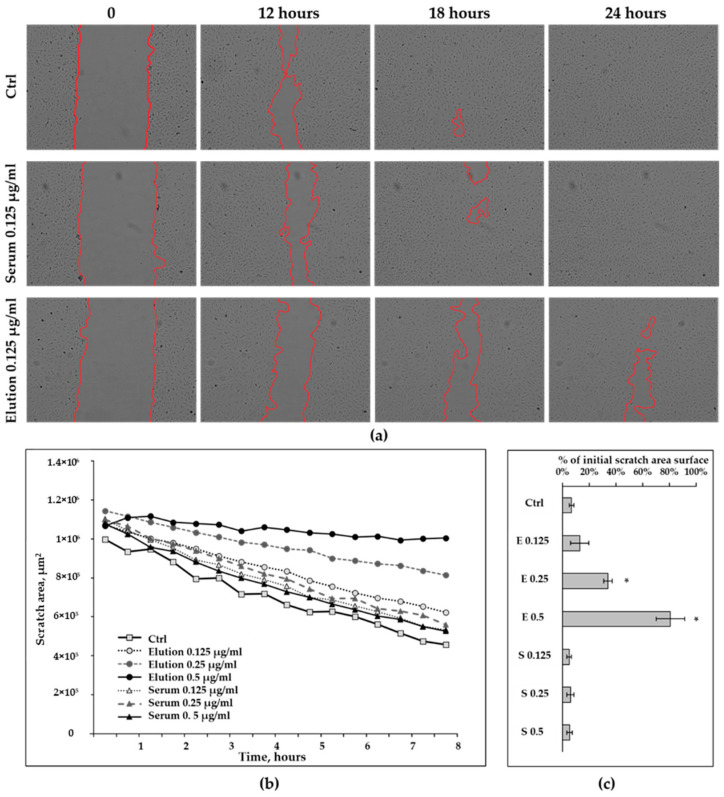
Wound-healing assay in HAECs treated with different concentration of proteins, eluted from the matrix of the hemoadsorber (E) and corresponding concentrations of human serum (S) (*n* = 3). (**a**) Representative microphotographs of the HAECs at different time points after scratch. Scratch borders are delineated in red; all microphotographs are taken with 4×magnification. (**b**) Dynamics of scratch area changes during the 8 h. (**c**) Percentage of initial scratch area surface after 24 h. Data are presented as mean ± SEM, * *p* < 0.05 (*t*-test) compared to the untreated control.

Complete closure of the scratch on a cell monolayer with a lateral dimension of approximately 100 µm normally succeeded after 18–20 h (Figure 2a). Cells treated with proteins eluted from the CytoSorb adsorbers used for intraoperative hemoadsorption demonstrated dose-dependent retardation in lateral migration during the first 8 h (scratch closure not attributed to cell division [25]) and beyond (Figure 2b,c). Complete closure of the scratch did not take place even with the lowest concentration of eluted proteins added to the culture medium (Figure 2a,c and Supplemental Figure S2). Scratch area in the cells treated with 0.25 µg/mL and 0.5 µg/mL of eluted proteins was significantly increased, compared to untreated control after 24 h (Figure 2c) and for the cells treated with the highest dose, the scratch area was changed by only 20% from initial scratch area surface after 24 h of observation. There was no significant difference in scratch closure between the serum-treated (S) and untreated controls (Figure 2c and Supplemental Figure S3).

### 3.3. Up-Regulated Genes in Treated HAECs

**Figure 3:**
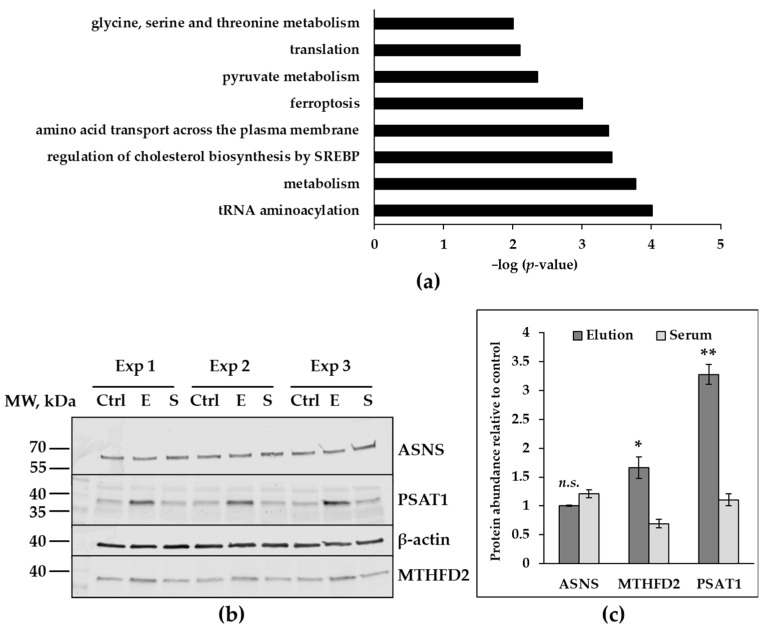
Targets involved into metabolism regulators, up-regulated in HAECs after 24 h treatment. (**a**) Biological process categories overrepresented in 201 up-regulated genes in HAECs after 24 h treatment with eluted proteins (array data, *n* = 2). Significance generated with ConsensusPathDB-human (release 34, [26]) is presented on the x-axis as negative logarithmized *p*-value. (**b**) Validation of up-regulated protein targets with immunoblotting. Proteins of interest were detected in cell lysates obtained from untreated HAECs (Ctrl), HAECs treated with 0.125 µg/mL of elution (E), 0.125 µg/mL of serum-control (S). Samples from three independent experiments are marked as Exp 1, Exp 2, Exp 3. (**c**) Signal intensities from protein targets were normalized to signal intensities from β-actin, signals from treated cells are expressed as percent from untreated control. The data are presented as mean values with ± SEM (* *p* ≤ 0.05; ** *p* ≤ 0.001; n.s.—no significant difference compared to untreated control). Abbreviations: ASNS−asparagine aminotransferase, PSAT1–phosphoserine aminotransferase, SREBP−sterol regulatory element-binding proteins; tRNA–transfer RNA, MTHFD2−methylene tetrahydrofolate dehydrogenase 2.

Microarray analyses revealed several heterogeneous groups of genes that were up-regulated in HAECs as a result of 24 h treatment with proteins eluted from the CytoSorb polymer matrix. Major groups, demonstrating significant enrichment in the set of up-regulated genes included genes involved in the regulation of tRNA aminoacylation and translation. Another major cluster of up-regulated genes was comprised of metabolic controllers, particularly regulators of amino acid, cholesterol, and pyruvate metabolism. Genes responsible for the transport of amino acids across the cell membrane were also overrepresented among up-regulated genes, as well as proteins involved into ferroptosis, a specific type of programmed cell death (Figure 3a). Among the 20 most up-regulated genes, clusters of genes encoding enzymes catalyzing amino acid biosynthesis, transcriptional regulators, and factors of growth and differentiation could be identified (Supplemental Table S3). To validate some of the data obtained from the mRNA-array, a number of targets were analyzed by Western blot. In contrast to asparagine aminotransferase, (ASNS) the protein levels of phosphoserine aminotransferase (PSAT1), and mitochondrial methylene tetrahydrofolate dehydrogenase 2 (MTHFD2) were significantly increased compared to untreated control (Figure 3b,c).

**Figure 4:**
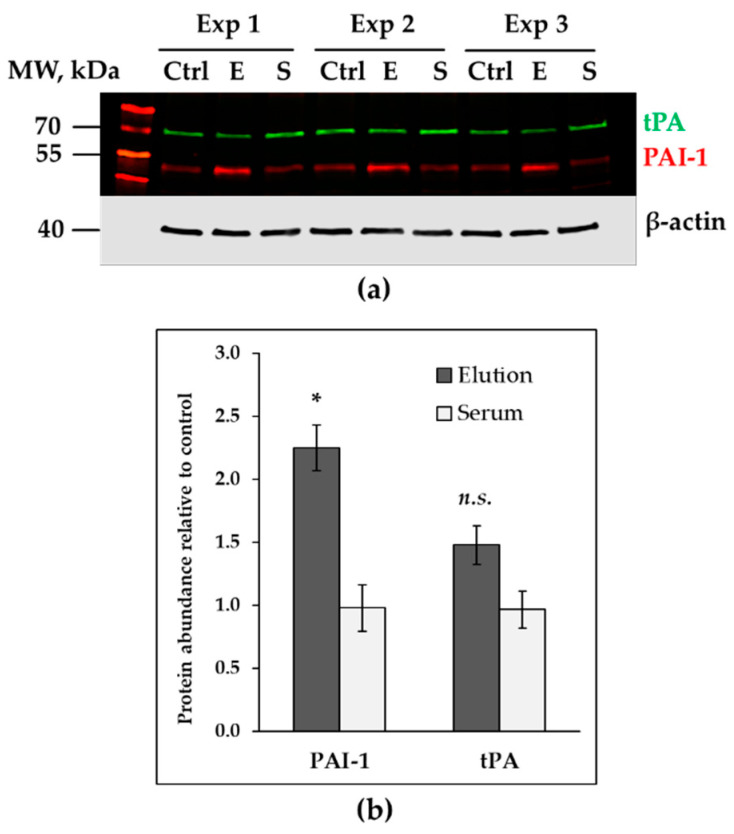
Increased expression of PAI-1 in HAECs, treated with proteins eluted from CytoSorb adsorber matrix (*n* = 3). (**a**) Immunodetection of tPA and PAI-1 in cell lysates obtained from untreated HAECs (Ctrl), HAECs treated with 0.125 µg/mL of elution €, 0.125 µg/mL of serum (S). Samples from three independent experiments are marked as Exp 1, Exp 2, Exp 3. (**b**) Signal intensities from PAI-1 and tPA, normalized to β-actin and expressed as fold signal intensity of an untreated control. Data are presented as mean ± SEM (* *p* ≤ 0.05, n.s.—no significant difference compared to controls).

Immunoblotting also confirmed an increase in plasminogen activator inhibitor 1 (PAI-1) levels in HAECs after exposure to proteins eluted from the adsorbers (Figure 4a,b). Protein expression of tissue plasminogen activator (tPA) did not significantly increase in response to 24 h treatment (Figure 4a,b); we also could not detect presence of tPA/PAI-1 complexes in cell lysates.

### 3.4. Down-Regulated Genes in Treated Endothelial Cells

**Figure 5:**
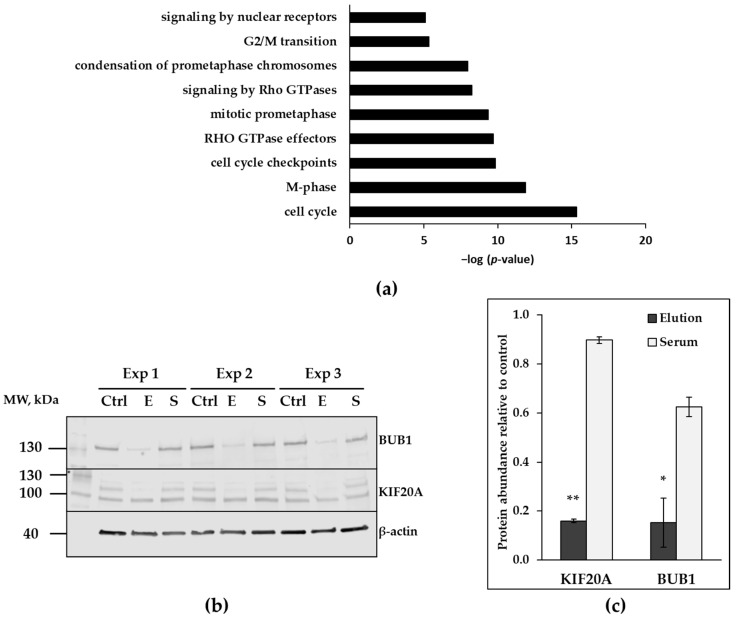
Targets involved into regulation of the cell cycle, down-regulated in HAECs after 24 h treatment with elution fraction. (**a**) Biological process categories overrepresented in 119 down-regulated genes in HAECs after 24 h treatment with eluted proteins (array data, *n* = 2). Significance generated with ConsensusPathDB-human (release 34, [26]) is presented on the x-axis as negative logarithmized *p*-value. (**b**) Validation of down-regulated protein targets with immunoblotting (*n* = 3). Proteins of interest were detected in cell lysates obtained from untreated HAECs (Ctrl), HAECs treated with 0.125 µg/mL of elution (E), 0.125 µg/mL of serum (S). Samples from three independent experiments are marked as Exp 1, Exp 2, Exp 3. KIF20A band is shown with the arrow. (**c**) Signal intensities from protein targets were normalized to signal intensity from β-actin, signals from treated cells are expressed as percent from untreated control. Data are presented as mean ± SEM. (* *p* ≤ 0.05; ** *p* ≤ 0.001).

Microarray data uncovered 119 genes down-regulated in HAECs after 24 h treatment with 0.125 µg/mL of proteins eluted from CytoSorb polymer matrix. Numerous genes are involved in cell cycle regulation (Figure 5a and Supplemental Table S4). The most down-regulated target according to mRNA-array data, kinesin family member 20A (KIF20A), was validated on the protein level. KIF20A, a mitotic protein essential for cell division is a key compound of the cytokinetic bridge, formed in the process of cell division into two daughter cells. KIF20A is phosphorylated by polo-like kinase 1 (PLK-1) and is recruited into the formation of the central spindle. KIF20A protein levels were significantly decreased in HAECs, treated with proteins eluted from the polymer matrix (Figure 5a,b). The phosphorylated form of this protein modified at the position Ser528 (phosphorylation site specific for PLK-1 phosphorylation) was also correspondingly reduced in cell lysates obtained from the cells treated with proteins recovered from adsorbers (Figure 5a).

Mitotic checkpoint kinase budding uninhibited by benzimidazoles 1 (BUB1) is another strongly down-regulated target upon treatment with protein mixture isolated from the polymer matrix identified by microarray. BUB1 is a serine/threonine protein kinase playing a key role in organization of mitotic spindle and chromosome alignment during the cell division. Consistently protein levels of BUB1 were significantly reduced in cell lysates derived from HAECs treated with eluted proteins (Figure 5b,c).

## 4. Discussion

The current study is the first providing a broad characterization of the blood proteins that are retained on the polymer matrix of hemoadsorbers during intraoperative hemoadsorption. Previous in vitro studies showed that CytoSorb adsorbers retain proteins in the size range between 5 and 60 kDa [13]. Recent in vitro study by Harm et al., revealed significant total plasma protein and albumin reduction during experimental plasma perfusion for 8 h [27]. Reduction of total protein concentration was more than 30%. It is important to underline, that 1 L of human plasma was used for the hemoadsorption experiment with 300 mL volume cartridge, which does not correspond to the ratio between total perfused blood volume and volume of cartridge in clinical conditions. We observed that despite that the majority of the isolated proteins are below 60 kDa, a quarter of adsorbed proteins was larger than 100 kDa.

Previous attempts to characterize the influence of CytoSorb hemoadsorption on endothelial cells has demonstrated significant impairment of cell–cell contacts and cytoskeletal architecture in cultured ECs, subjected to the serum of a septic patient. These pathological changes were alleviated when serum was subjected to hemoadsorption with CytoSorb [28]. In a later study, the CytoSorb adsorber was obtained from a patient with postoperative sepsis; the polymer material was eluted and fractionated with HPLC. This study also identified significant amounts of albumin, advanced glycation end products, nucleic acids, oxidized nucleotides, and amyloid-like aggregates in eluted fractions. Obtained fractions were tested in microvascular ECs, where they have resulted in increased apoptotic cell death and reduced ATP production [29]. The current study aimed to identify molecules retained in the CytoSorb adsorbers and to elucidate the effects of the protein extracts eluted from the CytoSorb devices on endothelial cells in vitro. Further studies are warranted to explore the dynamics of protein removal in different clinical settings (preemptive vs. postoperative approach), as well as inter-individual variability of protein adsorption depending on biological variation in blood composition, applied colloid, crystalloid solutions, fresh frozen plasma, and other blood products.

The present study has a range of limitations. First, we did not know to which degree the isolated fraction contained substances other than identified proteins, or how these could affect ECs. These substances potentially include bile acids, heme and products of hemoglobin degradation, various drugs, including vasoactive drugs and antimicrobial agents. Second, previous studies have identified that blood cells can adhere to the surface of the polymer beads [30,31]. We cannot exclude that a certain amount of blood cells were adsorbed on the surface of CytoSorb beads, eluted into the protein mixture later, launching metabolic and functional alterations in the cultured ECs as described above. Third, our experimental approach also did not allow precise quantification for each individual protein retained by the adsorber. Since the obtained protein extracts are a complex protein mixture, it was not possible to identify a single protein or protein combination responsible for the damage to ECs described in the present study.

It has been shown that increased expression of MTHFD2 and PSAT1 is associated with enhanced metabolism and survival, and it is often evident in tumor ECs [32,33]. In our study, on the contrary, ECs displayed reduced survival and impaired functionality. Massive up-regulation across many metabolic regulators identified in the current study is rather compensatory and adaptive. Increase of ASNS expression is also consistent with better cell growth and proliferation and is of particular importance for cell sprouting and angiogenesis [34]. The discrepancy between microarray-assay and the Western blot analysis regarding the expression of ASNS could be explained by posttranscriptional and posttranslational regulation with either quick degradation of mRNA after synthesis or by rapid turnover of the synthesized enzyme [35,36]. We observed a reduction in mRNA and protein levels of common mitotic proteins, KIF20A and BUB1, in ECs exposed to eluted protein mixture. These proteins accumulate during G1 and S phases of the cell cycle [37,38]. We observed severe impairment of cell growth and viability in CTB and scratch assays, therefore significant down-regulation in mitotic proteins represents overall growth retardation and cell cycle impairment in ECs.

Increased expression of PAI-1 and not tPA illustrates the pro-coagulant shift in EC metabolism after exposure to protein extract eluted from adsorber. ECs can produce increased amounts of pro-coagulant and anti-fibrinolytic agents in response to a plethora of inflammatory and damaging stimuli [39]. PAI-1 acts as an inhibitor of tPA, being the key inhibitor of fibrinolysis. Increased PAI-1 levels were previously reported in patients undergoing surgery with CPB [40,41,42]. The PAI-1/tPA ratio is an integral indicator of balance between clotting and fibrinolytic systems and is being discussed as a potential tool to assess the risk of thrombosis and bleeding [43,44].

In summary, the results of our tests in cultured endothelial cells suggest that the removal of a broad spectrum of blood proteins via CytoSorb hemoadsorption is potentially beneficial. It might exert protective properties by tackling an endothelial compound of systemic inflammatory dysregulation in patients undergoing valve surgery with CPB in acute endocarditis.

## 5. Conclusions

CytoSorb hemoadsorbers when applied intraoperatively bind a broad spectrum of blood proteins. According to our data, proteins retained on the polymer matrix generally possess molecular weight below 60 kDa and are mainly annotated as residential plasma proteins. Material eluted from the CytoSorb matrix used for hemoadsorption in patients with infective endocarditis causes dose-dependent and significant reduction in viability and migratory capacity of cultured endothelial cells. Eluted proteins initiate massive transcriptional changes including compensatory up-regulation in key metabolic controllers, along with depletion in mitotic proteins. Production of pro-coagulant PAI-1 was significantly increased after ECs exposure to the eluted protein mixture. CytoSorb hemoadsorption can potentially prevent endothelial damage and pro-coagulant changes during CPB by binding and removal of a broad spectrum of circulating detrimental factors.

## Data Availability

The data presented in this study are available in the Supplementary Material.

## Supplementary Materials

The following supporting information can be downloaded at: https://www.mdpi.com/article/10.3390/healthcare11030310/s1, Figure S1: SDS-PAGE analysis of the eluted material, derived from CytoSorb cartridges used for intraoperative hemoadsorption. Figure S2: Wound-healing assay dynamics in HAECs treated with different concentration of eluted material. Figure S3: Wound-healing assay dynamics in HAECs treated with control human serum. Table S1: Antibodies for Western blotting; Table S2: Proteins isolated in all 11 elution samples (*n* = 127), their molecular weight and functional annotation; Table S3: Top-20 most significantly up-regulated genes in elution-treated HAECs (mRNA-array data, *n* = 2); Table S4: Top-20 most significantly down-regulated genes in elution-treated HAECs (mRNA-array data, *n* = 2); Table S5: Pre- and postoperative plasma biomarkers of inflammatory response in patients undergoing valve surgery with cardiopulmonary bypass and hemoadsorption; Table S6: Laboratory parameters of the blood in pre- and postoperative period.

## Abbreviations

BUB1	Mitotic checkpoint kinase
CPB	Cardiopulmonary bypass
CCB	Coomassie colloidal blue
ECGM2	Endothelial cell growth medium 2
ECs	Endothelial cells
FCS	Fetal calf serum
HAECs	Human aortic endothelial cells
KIF20A	Kinesin superfamily 20A
PAI-1	Plasminogen activator inhibitor 1
PBS	Phosphate-buffered saline
RIN	RNA integrity number
RIPA	Radioimmunoprecipitation assay buffer
SDS-PAGE	Sodium dodecyl sulfate polyacrylamide gel electrophoresis
SIRS	Systemic inflammatory response syndrome
SEM	Standard error of mean
TBS	Tris-buffered saline
tPA	Tissue plasminogen activator
