# Supplementary material for: Proteins Adsorbed during Intraoperative Hemoadsorption and Their In Vitro Effects on Endothelium
Source: Healthcare (Basel). 2023 Jan 19;11(3):310. doi: 10.3390/healthcare11030310 (PMC9914797; doi:10.3390/healthcare11030310)
Supplement: Supplementary file 1 [file healthcare-11-00310-s001.zip › healthcare-2070322-supplementary.pdf]

Supplemental data

Table S1. Antibodies for western blotting.

Target protein	1°Ab /2°Ab	Species	Manufacturer	Ref. Nr.	Dilution	Incubation conditions
β-actin	1°Ab	Rabbit	Cell Signalling Technology, Danvers, MA, USA	#8457	1:1000	1 hour, RT
β-actin (AC-15)	1°Ab	Mouse	Sigma Aldrich, St. Louis, MO, USA	A5441	1:1000	Overnight, 4°C
Checkpoint mitotic kinase (BUB1)	1°Ab	Rabbit	Abcam, Cambridge, United Kingdom	ab126254	1:1000	Overnight, 4°C
Goat anti-rabbit green	2°Ab	Goat	Li-Cor Biosciences, Lincoln, NE, USA	926-32211	1:20000	1 hour, RT, dark
Goat anti-mouse red	2°Ab	Goat	Li-Cor Biosciences, Lincoln, NE, USA	926-68020	1:20000	1 hour, RT, dark
Kinesin superfamily protein 20 (KIF20A)	1°Ab	Rabbit	Abcam, Cambridge, United Kingdom	ab70791	1:1000	Overnight, 4°C
Methylene-tetrahydrofolate reductase 2, mitochondrial (MTHFD2)	1°Ab	Rabbit	Abcam, Cambridge, United Kingdom	ab151447	1:1000	Overnight, 4°C
Plasminogen activator inhibitor 1 (PAI-1)	1°Ab	Mouse	Santa Cruz Biotechnology, Dallas, TX, USA	sc-5297	1:500	Overnight, 4°C
Phosphoserine aminotransferase- 1 (PSAT1)	1°Ab	Rabbit	Abcam, Cambridge, United Kingdom	ab96136	1:1000	Overnight, 4°C
Tissue plasminogen activator (tPA)	1°Ab	Rabbit	Boster Bio, Pleasanton, CA, USA	PB9345	1:1000	Overnight, 4°C

1°Ab – primary antibody; 2°Ab – secondary antibody.

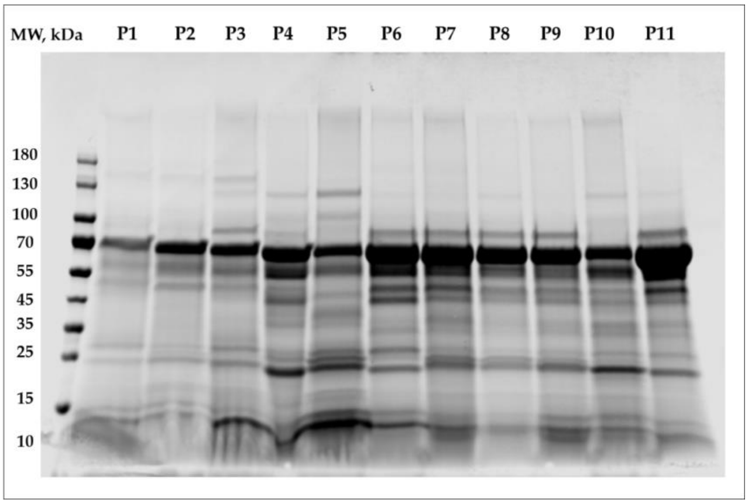

Figure S1. SDS-PAGE analysis of the eluted material, derived from CytoSorb cartridges used for intraoperative hemoadsorption. P1-P6 represent patient samples derived from the filters used for IE surgery, P7-P11 – samples eluted from the filters used during elective valve surgery. Each lane contains 20 µg of protein, gel stained with CCB and visualized with Li-Cor Odyssey scanner at 700 nm wavelength.

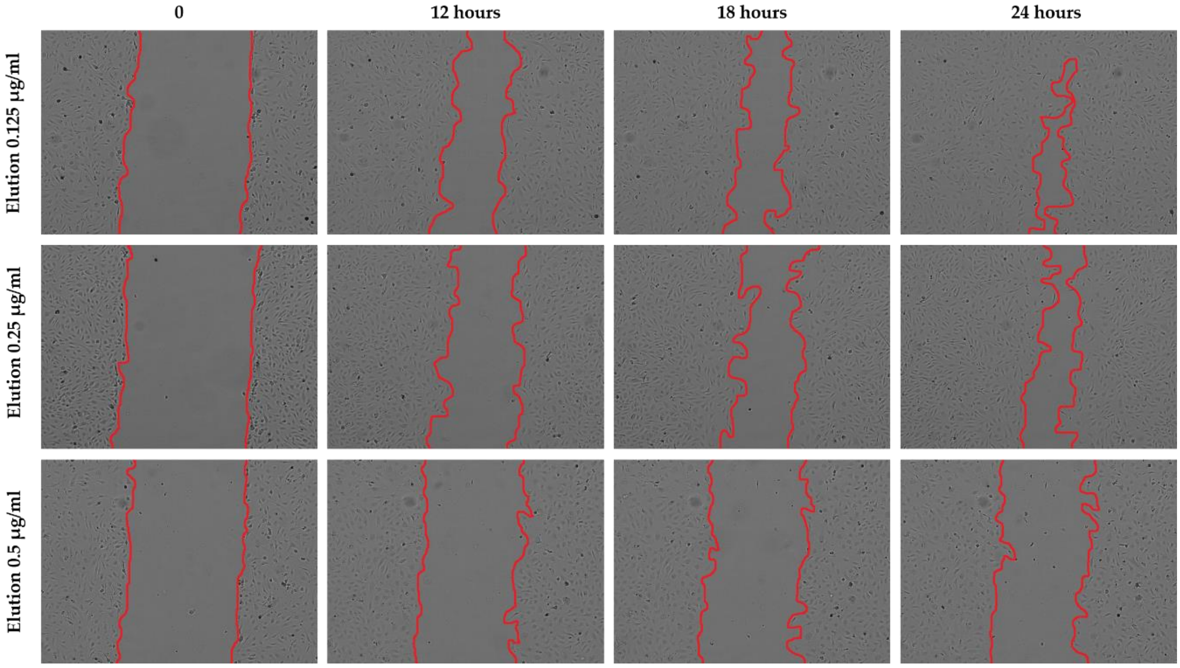

Figure S2. Wound healing assay dynamics in HAECs treated with different concentration of eluted material.

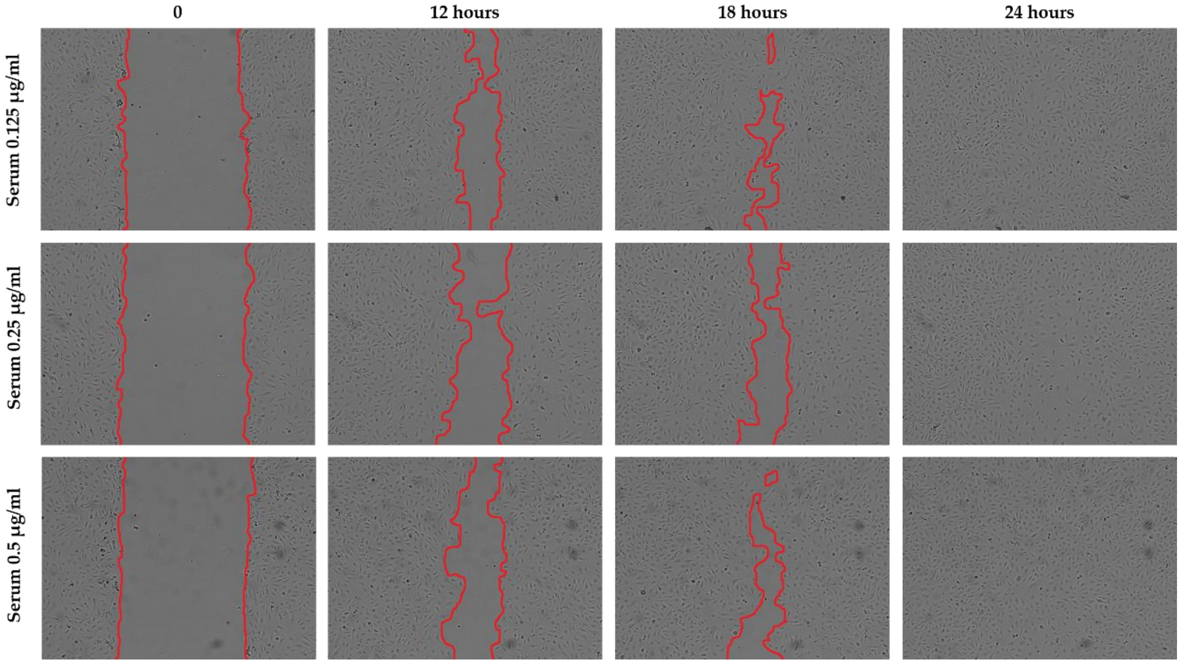

Figure S3. Wound healing assay dynamics in HAECs treated with control human serum.

Table S2. Proteins isolated in all 11 elution samples (n=127), their molecular weight and functional annotation.

UniProt ID	Gene name	Protein name	MW, kDa
P02768	ALB	Serum albumin	69.4
P21333	FLNA	Filamin-A	280.7
P01024	C3	Complement C3	187.1
P02788	LTF	Lactotransferrin (Lactoferrin)	78.2
P02787	TF	Serotransferrin (Transferrin)	77.1
P02774	GC	Vitamin D-binding protein	52.9
P00747	PLG	Plasminogen	90.6
P00450	CP	Ceruloplasmin	122.2
P02751	FN1	Fibronectin	272.3
P11215	ITGAM	Integrin alpha-M	127.2
Q14624	ITIH4	Inter-alpha-trypsin inhibitor heavy chain H4	103.4
P01042	KNG1	Kininogen-1	72
P01009	SERPINA1	Alpha-1-antitrypsin	46.7
P08603	CFH	Complement factor H	139.1
P06727	APOA4	Apolipoprotein A-IV	45.4
P02749	APOH	Beta-2-glycoprotein 1	38.3
P05107	ITGB2	Integrin beta-2	84.8
P35579	MYH9	Myosin-9	226.5
P04196	HRG	Histidine-rich glycoprotein	59.6
P01011	SERPINA3	Alpha-1-antichymotrypsin	47.7
P10909	CLU	Clusterin	52.5
P05106	ITGB3	Integrin beta-3 (Platelet membrane glycoprotein IIIa)	87.1
P00751	CFB	Complement factor B	85.5
P02647	APOA1	Apolipoprotein A-I	30.8
P08514	ITGA2B	Integrin alpha-IIb	113.4
P02790	HPX	Hemopexin	51.7
P14780	MMP9	Matrix metalloproteinase-9	78.5
P02671	FGA	Fibrinogen alpha chain	94.9

P02675	FGB	Fibrinogen beta chain	55.9
P18206	VCL	Vinculin	123.8
P02753	RBP4	Retinol-binding protein 4	23.0
P27105	STOM	Erythrocyte band 7 integral membrane protein	31.7
P02679	FGG	Fibrinogen gamma chain	51.5
P18065	IGFBP2	Insulin-like growth factor-binding protein 2	34.8
P02765	AHSG	Alpha-2-HS-glycoprotein	39.3
P18428	LBP	Lipopolysaccharide-binding protein (LBP)	53.4
P08697	SERPINF2	Alpha-2-antiplasmin	54.6
P01008	SERPINC1	Antithrombin-III	52.6
P08575	PTPRC	Receptor-type tyrosine-protein phosphatase C	147.5
P0DOX5	IGHG1	Immunoglobulin gamma-1 heavy chain	49.3
P04217	A1BG	Alpha-1B-glycoprotein	54.3
P00734	F2	Prothrombin	70.0
P04004	VTN	Vitronectin	54.3
P80188	LCN2	Neutrophil gelatinase-associated lipocalin	22.6
P00738	HP	Haptoglobin	45.2
P43652	AFM	Afamin	69.1
P17213	BPI	Bactericidal permeability-increasing protein	53.9
P05160	F13B	Coagulation factor XIII	75.5
P03950	ANG	Angiogenin	16.6
P24158	PRTN3	Myeloblastin	27.8
P02763	ORM1	Alpha-1-acid glycoprotein 1	23.5
Q16610	ECM1	Extracellular matrix protein 1	60.7
P01876	IGHA1	Immunoglobulin heavy constant alpha 1	37.7
P61769	B2M	Beta-2-microglobulin	13.7
Q12805	EFEMP1	EGF-containing fibulin-like extracellular matrix protein 1	54.6
P39060	COL18A1	Collagen alpha-1(XVIII) chain	178.2
P01834	IGKC	Immunoglobulin kappa constant	11.8
P05156	CFI	Complement factor I	65.8
P01859	IGHG2	Immunoglobulin heavy constant gamma 2	35.9
P0DJI8	SAA1	Serum amyloid A-1 protein	13.5
P0DJI9	SAA2	Serum amyloid A-2 protein	13.5
P08246	ELANE	Neutrophil elastase	28.5
P02775	PPBP	Platelet basic protein	13.9
P16284	PECAM1	Platelet endothelial cell adhesion molecule	82.5
P16671	CD36	Platelet glycoprotein 4	53.1
P06396	GSN	Gelsolin	85.7
P22692	IGFBP4	Insulin-like growth factor-binding protein 4	27.9
P68871	HBB	Hemoglobin subunit beta	15.9
P11021	HSPA5	Endoplasmic reticulum chaperone BiP	72.3
P00746	CFD	Complement factor D	27.0
P05556	ITGB1	Integrin beta-1	88.4
Q9Y490	TLN1	Talin-1	269.8
P05109	S100A8	Protein S100-A8	10.8
P19652	ORM2	Alpha-1-acid glycoprotein 2	23.6
P60709	ACTB	Actin, cytoplasmic 1	41.7
P06702	S100A9	Protein S100-A9	13.2
P49913	CAMP	Cathelicidin antimicrobial peptide	19.3
P07996	THBS1	Thrombospondin-1	129.4
P01860	IGHG3	Immunoglobulin heavy constant gamma 3	41.3

P61626	LYZ	Lysozyme C	16.5
P34096	RNASE4	Ribonuclease 4	16.8
P0DOY2	IGLC2	Immunoglobulin lambda constant 2	11.3
P40199	CEACAM6	Carcinoembryonic antigen-related cell adhesion molecule 6	37.2
P11279	LAMP1	Lysosome-associated membrane glycoprotein 1	44.9
Q9Y277	VDAC3	Voltage-dependent anion-selective channel protein 3	30.7
P31997	CEACAM8	Carcinoembryonic antigen-related cell adhesion molecule 8	38.2
P05452	CLEC3B	Tetranectin	22.5
P45880	VDAC2	Voltage-dependent anion-selective channel protein 2	31.6
P07359	GP1BA	Platelet glycoprotein Ib alpha chain	71.5
P05546	SERPIND1	Heparin cofactor 2	57.1
P01034	CST3	Cystatin-C	15.8
P01861	IGHG1	Immunoglobulin heavy constant gamma 4	35.9
P02760	AMBP	Protein AMBP	38.9
P00748	F12	Coagulation factor XII	67.8
P0C0L4	C4A	Complement C4-A	192.8
P04839	CYBB	Cytochrome b-245 heavy chain	65.3
P02748	C9	Complement component C9	63.2
P0DOX7	IGKV1-5	Immunoglobulin kappa light chain	23.4
P13498	CYBA	Cytochrome b-245 light chain	21.0
Q03591	CFHR1	Complement factor H-related protein 1	37.7
P16109	SELP	P-selectin	90.1
P21796	VDAC1	Voltage-dependent anion-selective channel protein 1	30.8
P06681	C2	Complement C2	83.3
P62979	UBA52	Ubiquitin-40S ribosomal protein S27a	17.9
P04179	SOD2	Superoxide dismutase [Mn], mitochondrial	24.8
P43304	GPD2	Glycerol-3-phosphate dehydrogenase, mitochondrial	80.9
P02654	APOC1	Apolipoprotein C-I	9.3
P02656	APOC3	Apolipoprotein C-III	10.9
P59665	DEFA1	Neutrophil defensin 1	10.2
P27824	CANX	Calnexin	67.6
Q99969	RARRES2	Retinoic acid receptor responder protein 2	18.6
P24592	IGFBP6	Insulin-like growth factor-binding protein 6	25.3
Q08722	CD47	Leukocyte surface antigen CD47	35.2
P21926	CD9	CD9 antigen	25.4
P05090	APOD	Apolipoprotein D	21.3
Q16627	CCL14	C-C motif chemokine 14	10.7
Q9Y624	F11R	Junctional adhesion molecule A	32.6
P61224	RAP1B	Ras-related protein Rap-1b	20.8
P13473	LAMP2	Lysosome-associated membrane glycoprotein 2	44.9
P13224	GP1BB	Platelet glycoprotein Ib beta chain	21.7
O14773	TPP1	Tripeptidyl-peptidase 1	61.2
P13796	LCP1	Plastin-2	70.3
P02042	HBD	Hemoglobin subunit delta	16.1
P30048	PRDX3	Thioredoxin-dependent peroxide reductase, mitochondrial	27.7
P55774	CCL18	C-C motif chemokine 18	9.8
P08571	CD14	Monocyte differentiation antigen CD14	40.1
P36980	CFHR2	Complement factor H-related protein 2	30.7

Table S3. Top-20 most significantly up-regulated genes in elution-treated HAECs (mRNA-array data, n=2).

Gene name	RefSeq ID	Protein name	Fold up-regulated	p-value
PSAT1	NM_021154	Phosphoserine aminotransferase 1	18.30	0.005
ASNS	NM_001178075	Asparagine synthetase (glutamine-hydrolyzing)	15.79	0.02
NUPR1	NM_001042483	Transcriptional regulator 1 (p8)	9.76	0.004
MTHFD2	NM_006636	Methylenetetrahydrofolate dehydrogenase (NADP+ dependent) 2	7.14	0.02
STC1	NM_003155	Stanniocalcin 1	6.47	0.04
DHCR7	NM_001163817	7-dehydrocholesterol reductase	6.31	0.002
SLC7A11	NM_014331	Solute carrier family 7	6.13	0.03
PTX3	NM_002852	Pentraxin 3, long	5.78	0.02
RAC2	NM_002872	Ras-related C3 botulinum toxin substrate 2	4.60	0.003
VEGFA	NM_001025366	Vascular endothelial growth factor A	4.57	0.03
SLC3A2	NM_001012662	Solute carrier family 3 (amino acid transporter heavy chain), member 2	4.49	0.003
ERRFI1	NM_018948	ERBB receptor feedback inhibitor 1	4.43	0.02
ANKRD20A3	NM_001012419	Ankyrin repeat domain 20 family, member A3	4.42	0.007
NOB1	NM_014062	NIN1/RPN12 binding protein 1 homolog	4.36	0.002
SARS	NM_006513	Seryl-tRNA synthetase	4.27	0.04
ZCCHC7	NM_001289119	Zinc finger, CCHC domain containing 7	4.22	0.04
STC2	NM_003714	Stanniocalcin 2	4.18	0.04
MYADM	NM_001020818	Myeloid-associated differentiation marker	4.14	0.002
ZBTB41	NM_194314	Zinc finger and BTB domain containing 41	4.00	0.04
TGFB1I1	NM_001042454	Transforming growth factor beta 1 induced transcript 1	4.00	0.002

Abbreviations: BTB - Broad-Complex, Tramtrack and Bric a brac; CCHC – CysCysHisCys domain; ERBB - Receptor tyrosine-protein kinase erbB-2; GTP – guanosintriphosphate; NADP+ - nicotinamide adenine dinucleotide phosphate; NIN1 - Neutral/alkaline invertase 1; Ras – rat sarcoma gene, encoding small G-protein; RPN12 - proteasome regulatory particle lid subunit; tRNA – transfer RNA.

Table S4: Top-20 most significantly down-regulated genes in elution-treated HAECs (mRNA-array data, n=2).

Gene name	RefSeq ID	Protein name	Fold down-regulated	p-value
KIF20A	NM_005733	Kinesin family member 20A	0.13	0.01
PBK	NM_001278945	PDZ binding kinase	0.15	0.01
FABP5	NM_001444	Fatty acid binding protein 5 (psoriasis-associated)	0.15	0.04
TXNIP	NM_006472	Thioredoxin interacting protein	0.16	0.01
CENPI	NM_006733	Centromere protein I	0.17	0.01
FOXM1	NM_001243088	Forkhead box M1	0.18	0.01
DLGAP5	NM_001146015	Discs, large (Drosophila) homolog-associated protein 5	0.18	0.04
PRC1	NM_001267580	Protein regulator of cytokinesis 1	0.19	0.04
GINS1	NM_021067	GINS complex subunit 1 (Psf1 homolog)	0.20	0.03
BUB1	NM_001278616	BUB1 mitotic checkpoint serine/threonine kinase	0.20	0.05
TPX2	NM_012112	TPX2, microtubule-associated	0.21	0.01
CCNB1	NM_031966	Cyclin B1	0.22	0.004
EFNB2	NM_004093	Ephrin-B2	0.23	0.004
FAM83D	NM_030919	Family with sequence similarity 83, member D	0.24	0.05
NDC80	NM_006101	NDC80 kinetochore complex component	0.24	0.04
ESCO2	NM_001017420	Establishment of sister chromatid cohesion N-acetyltransferase 2	0.24	0.03
PCDH10	NM_020815	Protocadherin 10	0.24	0.01
DIAPH3	NM_001042517	Diaphanous-related formin 3	0.25	0.04
MGP	NM_000900	Matrix Gla protein	0.25	0.04
ASPM	NM_001206846	Abnormal spindle microtubule assembly	0.25	0.02

Abbreviations: BUB1 - budding uninhibited by benzimidazoles 1; GINS - an acronym created from the first letters of the Japanese numbers 5-1-2-3 (*go-ichi-ni-san*) in a reference to the 4 protein subunits of the complex: Sld5, Psf1, Psf2, and Psf3; Gla - vitamin K-dependent carboxylation/gamma-carboxyglutamic domain; NDC80 - kinetochore complex component; PDZ (initialism) – protein domain common for post-synaptic density protein (PSD95), Drosophila disc large tumor suppressor (Dlg1), and zonula occludens-1 protein (zo-1); Psf1 – protein of GINS-complex; TPX2 - microtubule nucleation factor.

Table S5. Pre- and postoperative plasma biomarkers of inflammatory response in patients undergoing valve surgery with cardiopulmonary bypass and hemoadsorption.

Patient number	C-reactive protein, mg/l		Procalcitonin, μg/l	
	Pre	Post	Pre	Post
1	80	64	3.83	0.42
2	54.9	37.4	8.53	92.9
3	166	51	0.12	0.71
4	6	49	0.03	8.06
5	41	71	1.14	1.82
6	48	29	0.5	1.76
7	34	92	n.a.	0.84
8	13	54	n.a.	1.8
9	2.3	101	n.a.	1.95
10	4.4	76.5	n.a.	0.51
11	5.7	48.7	n.a.	0.19

Table S6. Laboratory parameters of the blood in pre- and postoperative period.

Patient number	Total plasma protein, g/l		Albumin, g/l		Fibrinogen, g/l		Hb, g/dL		Er, 10^{12}/l		HCT, l/l	
	Pre	Post	Pre	Post	Pre	Post	Pre	Post	Pre	Post	Pre	Post
1	n.a.	48	n.a.	27.1	4.31	3.9	4	5.1	2.52	3.08	0.19	0.24
2	78	61‡	27.8	7.8	3.41	2.28	7.2	6.7	4.59	4.13	0.36	0.32
3	67	28	28	11	3.59	3.04	5.8	5.4	4.2	3.2	0.32	0.23
4	62	49	35.6	26	4.53	4.09	8.7	7.7	4.46	3.19	0.40	0.35
5	58‡	56	22‡	20.6	n.a.	3.18	4.4	7.6	2.46	4.39	0.21	0.36
6	79‡	48	45.3‡	24,2	3.9	2.56	4.5	6.6	2.4	3.5	0.22	0.3
7	80	46	n.a.	29.5	5.99	3.41	7.4	5.9	3.63	3.04	0.34	0.27
8	79	45	n.a.	21.4	4.27	3.67	7.4	6.4	3.86	3.35	0.35	0.3
9	76	45	n.a.	34.9	3.25	3.41	9.6	6.9	5.1	3.67	0.43	0.31
10	58	37	36.6	29.5	4.4	3.77	8.6	5.3	4.87	2.96	0.42	0.26
11	76	51	n.a.	29.7	4.45	2.98	7	5.3	3.95	2.88	0.34	0.25

Abbreviations: Hb – hemoglobin; Er – red blood cells; HCT – hematocrite. n.a. – some of the parameters were not routinely measured in preoperative settings; ‡ – marked parameters were not measured directly in pre-operative settings.
